# Research progress on macrophage regulation of atherosclerosis

**DOI:** 10.3389/fcvm.2026.1682994

**Published:** 2026-02-04

**Authors:** Jiahui Song, Rui Yan, Ping Li, Jincheng Guo, Guangyao Zhai, Jing Li

**Affiliations:** 1 Department of Cardiology, Beijing Luhe Hospital, Capital Medical University, Beijing, China; 2 Department of Geriatrics, Xuanwu Hospital Capital Medical University, National Clinical Research Center for Geriatric Diseases, Beijing, China

**Keywords:** atherosclerosis, macrophages, inflammatory response, efferocytosis, therapeutic advances

## Abstract

Cardiovascular diseases (CVDs) currently are responsible for high disability and mortality rates worldwide. Atherosclerosis is a major pathological process leading to CVDs, and macrophages are known to be important contributors to the initiation of atherosclerosis and promotion of plaque development, which eventually leads to plaque rupture. The role of these cells as a driver of plaque instability has received widespread attention, with continued research efforts devoted to unraveling the underlying mechanisms of macrophage involvement in atherosclerosis and identifying specific therapeutic strategies. In this review, we summarize the current evidence regarding the mechanisms by which macrophages regulate the progression of plaque development as well as recent therapeutic advances for the treatment and prevention of atherosclerosis.

## 1 Introduction

With the continuous development and optimization of vascular reconstruction techniques, standardized drug treatments, and prevention strategies, the effectiveness of clinical treatments for atherosclerosis and its complications has greatly improved. However, the unpredictable rupture of vulnerable atherosclerotic plaques can still lead to adverse cardiovascular events, such as acute coronary syndrome and even sudden cardiac death. The incidence rate of such events has continued to rise in the past 30 years and remains the leading cause of disease burden worldwide (1). Currently, atherosclerosis is attributed to chronic aseptic inflammation, and macrophages, as the main immune cells involved in atherosclerosis, play important roles at different stages of the disease. Histological studies of plaques from patients who died of acute coronary artery disease confirmed the presence of a large necrotic core, thin fibrous cap, and high proportions of macrophages and vascular smooth muscle cells (SMCs) (2) within the culprit lesion, indicating that vascular inflammation is closely related to plaque vulnerability. Macrophages accumulate locally and phagocytose oxidized modified lipoproteins to form foam cells. The resulting accumulation of foam cells leads to the formation of lipid-rich plaques, while the secretion of pro-inflammatory and chemotactic factors along with the generation of reactive oxygen species serve to maintain local inflammatory responses (3). With the progression of the lesion, the ability of macrophages to migrate within the plaque decreases, blocking effective extracellular excretion of waste and causing accumulation of apoptotic cells, ultimately resulting in the formation of the lipid necrotic core within atherosclerotic plaques (4–6). With the development of this necrotic core, the lesion becomes a complex and vulnerable plaque. Activated macrophages release metalloproteinases, which degrade collagen, and in the context of the inflammatory environment, also promote the thinning and even rupture of the fibrous cap, leading to exposure of the necrotic core and thrombosis (7, 8), which can induce fatal acute myocardial infarction (9). Thus, macrophages play crucial roles in the progression and regression of atherosclerotic lesions, prompting the need for in-depth exploration of the underlying mechanisms of these roles (1, 10).

Intravascular optical coherence tomography (OCT) is a technique that can provide near-pathological results, with a resolution of 10 µm. The application of OCT in clinical practice has enabled *in vivo* studies of local vascular inflammation in patients with acute coronary syndrome (ACS). The Coronary Lesion Morphology and Clinical Outcomes Relationship with OCT (CLIMA) study (NCT02883088) defined four characteristics of high-risk plaques: minimum lumen area <3.5 mm^2^, fibrous cap thickness FCT <75 mm, maximum lipid arc >180°, and macrophage aggregation (11). Macrophage infiltration is closely related to systemic inflammation, and two-thirds of ACS patients show plaque rupture on OCT examination, with extensive macrophage infiltration within plaques and elevated levels of systemic inflammatory biomarkers (12). One study in ACS patients with plaque rupture found that macrophages act to trigger thrombosis and inflammation (13). Consistently, another study in ACS patients with plaque rupture showed that there are usually more macrophages and microvessels in the local culprit vs. non-culprit plaques, indicating more severe local inflammation and suggesting a higher level of panvascular instability (14). Therefore, macrophage infiltration is an important initiating and promoting factor for atherosclerotic plaques.

### 1.1 Macrophage phenotype and function

**Figure 1:**
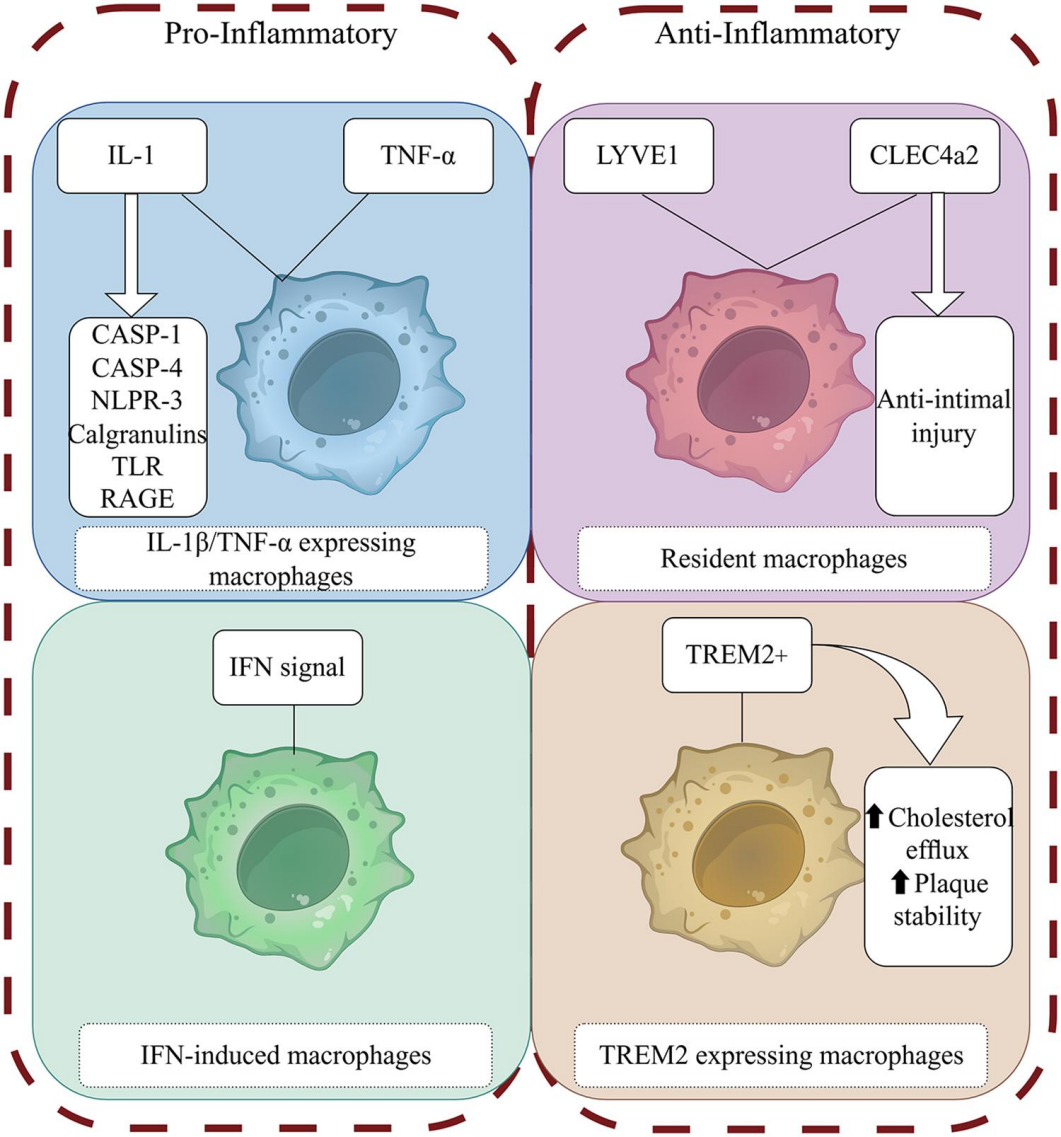
Macrophage phenotype and function. (By Figdraw) The figure illustrates the roles of different macrophage phenotypes in atherosclerosis. These phenotypes exert their effects through proinflammatory and proresolving mediators binding to specific receptors and activation of intracellular signaling pathways.

Most studies of the metabolic response of macrophages to inflammatory stimuli have focused primarily on two activation phenotypes: “pro-inflammatory” M1 macrophages stimulated by lipopolysaccharide (LPS) and interferon-γ (IFN-γ), known as classical macrophages, and “anti-inflammatory” M2 macrophages stimulated by interleukin-4 (IL-4), also known as alternatively activated macrophages (15). Although understanding how macrophage activation and metabolic responses are related under different inflammatory stimuli is useful, the classification of macrophages into the M1 and M2 polarization types is overly simplistic. Single-cell sequencing of samples from humans and mice with atherosclerosis has shown that the main macrophage populations include two types of pro-inflammatory macrophages: tissue-resident macrophages that express triggering receptor expressed on myeloid cells 2 (TREM2) macrophages and IFN-induced macrophages (IFNICs) (16–18) (Figure 1).

#### 1.1.1 IL-1β/TNF-α–expressing macrophages

Macrophage populations associated with atherosclerosis express the cytokine IL-1β (19) and the components needed for IL-1β generation, such as caspases 1–4 and NOD-like receptor 3 (NLRP3), as well as other crucial inflammatory mediators associated with atherosclerosis, such as the S100 calgranulins (S100A8/S100A9/S100A12), inflammation-induced Toll-like receptors (TLRs), and advanced glycation end products ligand receptors. At the same time, another subgroup of macrophages expresses tumor necrosis factor α (TNF-α), indicating heterogeneity in the inflammatory environment of plaques, to a certain degree (20), while still showing a strong pro-inflammatory gene signature.

#### 1.1.2 Resident macrophages

Tissue-resident macrophages derived from embryonic development can self-renew and maintain tissue balance (21). Resident macrophages in the mouse aorta have significant heterogeneity (22). In mouse studies, vascular resident lymphatic vessel endothelial hyaluronan receptor 1 (LYVE1)-expressing macrophages maintain vascular integrity and contractile function by regulating SMC function and controlling the balance of extracellular matrix production and degradation (23). In contrast, vascular resident C-type lectin domain family 4, member a2 (CLEC4a2)-expressing macrophages have been shown to have an anti-intimal injury effect (24). Previous research has suggested that resident macrophages play a role in maintaining vascular homeostasis and have anti-atherosclerotic functions (20).

#### 1.1.3 TREM2-expressing macrophages

The TREM2-positive macrophages identified by Kim et al. through Bodipy staining are foam cells (25), which are classified as lipid-associated macrophages (LAMs) (26) and exhibit lipid-processing capacity as well as express specific genes that regulate cholesterol metabolism and efferocytosis (25, 26). However, these cells do not produce any inflammatory cytokines and chemokines specific to atherosclerosis (18). The lack of gene expression related to inflammation challenges the notion of lipid-promoting inflammation (20), and these cells show a unique anti-inflammatory transcriptional signature (17). TREM2 + macrophages also are associated with the promotion of fibrosis (27). Accordingly, TREM2 + macrophages may be related to plaque stability (20). Studies have shown that TREM2 is related to cholesterol metabolism (28–31), and its possible mechanism involves the regulation of downstream apolipoprotein E (ApoE) expression (32). TREM2 plays a crucial role in regulating macrophage-mediated processes such as phagocytosis, inflammation and fibrosis resolution. However, the molecular mechanisms by which TREM2-expressing macrophages influence the progression of atherosclerosis remain incompletely understood.

#### 1.1.4 IFN-induced macrophages

IFN-induced macrophages (interferon-inducible cells, IFNICs) have been detected only in the aortas of atherosclerotic mice by single-cell RNA sequencing technology (18). This localization is similar to that of macrophages in ischemic hearts as described by King et al. (33), and a possible explanation is that the macrophages may be derived from monocytes and progenitor cells in the bone marrow with mediation by type I IFN after tissue injury (34). It remains unclear whether IFNICs play a balancing role or a strictly disease-inducing role. Given the atherogenic effect of IFN signal transduction (35), this cell cluster may be related to disease progression.

### 1.2 Macrophage-mediated inflammatory response

#### 1.2.1 NLRP3/IL-1β

Studies have demonstrated that a lipid peroxidation product, octanal, can activate the NLRP3 inflammasome and activate the expression of *olfactory receptor 2* (*Olfr2*) in mouse and the human homologous gene *OR6A2* in vascular macrophages, which together with TLR4, induce the production and secretion of IL-1α and IL-1β proteins, exacerbating atherosclerosis (36). Multiple studies have confirmed that IL-1β-induced activation of macrophages depends on the NLRP3 inflammasome, which is considered an indicator of metabolic inflammation and a central relay in the process of atherosclerosis (37, 38). However, blocking NLRP3 can have pleiotropic and immunological side effects, and thus, selectively targeting upstream stimulants is more feasible. However, this requires a deeper understanding of the immune regulatory pathways for atherosclerosis, with research efforts continuously searching for alternative inflammatory targets and more precise chronic treatment methods with fewer side effects (39).

The Canakinumab Anti-inflammatory Thrombosis Outcome Study (CANTOS) trial showed that systemic inhibition of IL-1β can effectively reduce inflammation and the recurrence of cardiovascular events (19), as well as control the residual risk caused by the inflammatory response, which is related to the activation of the NLRP3 inflammasome-driven IL-1β/IL6 pathway. Notably, inhibiting IL-1β can increase IL-4 levels and stimulate the proliferation and polarization of local macrophages toward the anti-inflammatory type (40). However, animal experiments have also shown that inhibiting IL-1β regulates the anti-inflammatory phenotype of resident macrophages in the fibrous cap, reduces the proliferation of SMCs, and may induce an illusion of inflammation resolution (40), leading to premature dissolution of the SMC/collagen-rich fibrous cap. Importantly, thin-cap fibroatheroma (TCFA) is considered an unstable high-risk plaque with a tendency to rupture (2). This seems to be inconsistent with the results of the CANTOS trial, The IL-1β signaling pathway is a classic pro-inflammatory pathway, while it plays a dual role in atherosclerosis, under specific pathological conditions, it may exhibit protective/regulatory functions, and thus, in-depth exploration of strategies to control inflammation are needed.

#### 1.2.2 PPAR-γ/TLR4/NF-κB

Peroxisome proliferator-activated receptor γ (PPARγ) agonists have anti-inflammatory and anti-atherosclerotic effects (41). Macrophage mannose receptor (MMR) targeting using mannose-modified chitosan-loaded with PPARγ agonist lobeglitazone to prepare nanoprobes (MMR-Lobe) was shown to effectively reduce plaque burden by activating cholesterol efflux in macrophage foam cells (42), inhibiting the TLR4/nuclear factor κB (NF-κB) pathway, increasing the expression of *ABCA1* mRNA, reducing the production of monocyte chemoattractant protein-1 (MCP-1), and reducing the amounts of macrophages and collagen-rich matrices, thereby exerting an anti-inflammatory effect. At the same time, this increases the collagen content, especially the type I collagen content, to increase the stability of the plaque (43).

#### 1.2.3 CD47/SIRPα/SHP-1

To delay the progression of atherosclerotic plaques, the phagocytosis of apoptotic debris in necrotic tissue is reactivated by blocking the CD47-signal regulatory protein α (SIRPα) signal axis. Targeted nanotechnology has been developed to apply the CD47-SIRPα inhibitor to macrophages within atherosclerotic plaques and shown to specifically regulate the CD47/SIRPα/SHP-1 (protein tyrosine phosphatase-1) axis, reducing the expression of pro-inflammatory cytokines and chemokines, stimulating phagocytes to clear apoptotic cells, inhibiting plaque progression. and reducing inflammation. Additionally, the phagocytic property of macrophages can minimize toxicity in other systems and reduce off-target clearance of healthy tissues (40).

#### 1.2.4 CD40/TRAF6

The immune metabolism of the body can change the inflammatory process, and research on the related targets has gradually led to a new direction in the treatment of atherosclerosis. The binding of CD40L to CD40 leads to the recruitment and signal transduction of TNF receptor-associated factors (TRAFs). The high expression of TRAF6 in macrophages is related to metabolic inflammation (44). CD40/TRAF6 signaling promotes atherosclerosis through the interaction of antigen-presenting cells (macrophages) and T cells (45), and blocking this pathway can reduce the production of pro-inflammatory cytokines such as TNF-α, IL-1β, and IL-6, while also effectively reducing the migration and activation of macrophages and rapidly reducing the infiltration of macrophages, thereby reducing atherosclerosis in mice and non-human primate models (46). These results demonstrate the translational potential of this strategy in the treatment of atherosclerosis. In addition, CD4+ T lymphocytes activate macrophages through the CD40/CD40 ligand signaling pathway (46), and CD4+ T cells lacking CD40L can attenuate atherosclerosis in a mouse model and affect T helper cell 1 (Th1) polarization and IFN-γ activation. The loss of CD40 signaling limits the occurrence of atherosclerosis and systemic inflammation by preventing macrophages from transforming along the polarization direction. Through the regulation of macrophage polarization, CD40 inhibitors targeting macrophages have been identified as a potentially valuable anti-atherosclerotic treatment strategy (47).

### 1.3 Macrophages and RNA regulation

Many of the cellular mechanisms, molecular factors, and inflammatory mediators of atherosclerosis have been elucidated, but the regulatory mechanisms of atherosclerosis involving microRNAs (miRNAs) remain incompletely understood. To date, research has identified approximately 2,300 true human mature miRNAs (48) that regulate more than 60% of human protein-coding genes (49) and have the potential to serve as therapeutic targets and biomarkers.

#### 1.3.1 miR-21/*Xaf1*

Interestingly, atherosclerotic plaque rupture and myocardial infarction are more likely to occur in the early morning (50, 51), and this has been linked to factors such as catecholamine surges, sympathetic nerve excitation, and elevated blood pressure. This observation also indicates that diurnal variations exist in the vulnerability of plaques. In atherosclerotic lesions, apoptosis peaks at the beginning of activity, and when the diurnal variations of exocytosis and apoptosis are inconsistent, the formation of the necrotic cores increases (52). miR21-5p is one of the most highly expressed miRNAs in macrophages and atherosclerotic lesions (53). *XIAP-associated factor-1* (*Xaf1*) is the only diurnally regulated gene that is upregulated after miR21 gene knockout in mouse atherosclerotic lesions. Expression of both miR21-5p and miR21-3p increases the survival rate of macrophages by inhibiting *Xaf1*-mediated activation of caspase 3 (54). In human atherosclerotic lesions, the anti-phase rhythm of miR-21 chain expression and *XAF1*-related apoptosis has also been confirmed. Therefore, the macrophage apoptosis clock controlled by miR21 may promote the growth and vulnerability of lesions, suggesting that the molecular clock can have harmful effects under pathological conditions (52).

#### 1.3.2 miR-155/BCL6/p-STAT-3

miR155 is specifically expressed in atherosclerotic plaques and pro-inflammatory macrophages. Previous studies have shown that deletion of miR155 reduces the expression of the chemokine CCL2, thereby promoting the recruitment of monocytes to atherosclerotic plaques (55). At the same time, increased expression of miR155 partially mediates its pro-atherogenic and pro-inflammatory effects by inhibiting the anti-inflammatory proteins B-cell lymphoma 6 (BCL-6) and phosphorylated signal transducer and activator of transcription 3 (p-STAT-3) in macrophages (56). miR155 directly inhibits the expression of BCL6, which is a transcription factor that attenuates pro-inflammatory NF-κB signaling (55). Another study showed that miR155-5p is regulated by conjugated linoleic acid (CLA). Linoleic acid is a mediator that promotes the alleviation of atherosclerosis. Dietary CLA was found to reduce the expression of miR155 in the aorta of apolipoprotein E-/- mice and thereby mediate the regression of atherosclerosis (56).

#### 1.3.3 NFATc3/miR204

Macrophages phagocytose oxidized low-density lipoprotein (oxLDL) to form foam cells, which are a hallmark of atherosclerosis (57, 58). oxLDL can activate the calcineurin/ apoptosis signal-regulating kinase 1 (ASK1)/ c-Jun N-terminal kinase (JNK) signaling pathway in macrophages, promoting the formation of foam cells and exacerbating atherosclerosis (59). Members of the nuclear factor of activated T cells (NFAT) family of proteins were first identified as transcription factors activated by Ca^2+^/calcineurin signaling in T cells (60). Research has since shown that overexpression of NFATc3 in macrophages inhibits the formation of foam cells (59). Additional studies have found that the level of NFATc3 in peripheral blood mononuclear cells of patients with atherosclerosis is negatively correlated with the instability of plaques. Furthermore, NFATc3 in macrophages inhibits the formation of foam cells and atherosclerosis by upregulating the cytoplasmic miR204-5p/SR-A and nuclear miR204-3p/CD36 axes and downregulating the scavenger receptor A (SR-A) and CD36 (61). At the same time, in the plaques of NFATc3-overexpressing mice, infiltration of macrophages and T cells is inhibited. Additionally, the levels of monocyte chemoattractant protein (MCP-1) and IL-1β in the mouse serum are significantly decreased, while the levels of IL-6 and IL-10 are increased. The current evidence indicates that NFATc3 can convert pro-inflammatory macrophages into macrophages with an anti-inflammatory phenotype (61), demonstrating that NFATc3 in macrophages is an important inhibitory factor for the occurrence of atherosclerosis.

#### 1.3.4 MIAT

A complete complementary deoxyribonucleic acid (cDNA) was isolated in a case-control association study of Japanese patients with myocardial infarction and named myocardial infarction-associated transcript (MIAT), also known as “Gomafu”. MIAT does not encode any proteins, suggesting that it is likely a functional RNA (62). Previous studies have demonstrated that long non-coding RNA (lncRNA)-MIAT, as a competitive endogenous RNA, forms a feedback loop with vascular endothelial growth factor and miR150-5p to regulate endothelial cell function and participate in diabetes-induced pathological angiogenesis and microvascular dysfunction (63).

In human carotid plaques and animal experiments, it has been further confirmed that MIAT promotes the proliferation of SMCs through the early growth response-1 (EGR1)/ETS transcription factor (ELK1)/extracellular signal-regulated kinase (ERK) pathway. MIAT further participates in the transformation of the SMC phenotype to pro-inflammatory macrophage-like cells by binding to the Kruppel-like factor 4 (KLF4) promoter and enhancing its transcription, and further activates pro-inflammatory macrophages through the NF-κB signaling pathway. Overall, the evidence indicates that lncRNA MIAT is a novel regulatory factor in the progression of atherosclerosis and plays a key functional role in the development and instability of atherosclerotic plaques, regulating cell proliferation, apoptosis, and the pro-inflammatory phenotype transformation of SMCs and macrophages (64).

### 1.4 Macrophages and efferocytosis

#### 1.4.1 LXRs

Activation of liver X receptors (LXRs) in macrophages can increase cholesterol efflux and control the inflammatory response by downregulating pro-inflammatory mediators in macrophages. In this process, high-density lipoprotein (HDL) acts as a receptor for cholesterol. To enhance reverse cholesterol transport (RCT), current preclinical studies include targeted delivery of LXR agonist nanoparticles to promote RCT and inflammatory responses, in order to reduce plaque macrophage inflammation and promote cholesterol efflux, while avoiding lipid accumulation in the liver. The results of these studies so far indicate that synthetic HDL-targeted nanoparticles loaded with LXR agonists offer a promising therapeutic approach (65, 66).

#### 1.4.2 EIMP

Macrophage clearance of apoptotic cells, a process known as efferocytosis, plays a central role in maintaining tissue homeostasis and controlling local inflammatory processes (67–69). Nucleotides derived from the hydrolysis of apoptotic cell DNA by phagolysosomal DNase2a activate the DNA-dependent protein kinase catalytic subunit (DNA-PKcs)/mammalian target of rapamycin complex 2 (mTORC2)/Rictor pathway, increase Myc expression, and promote the proliferation of non-inflammatory macrophages, which can induce the regression of atherosclerosis. This proliferation of macrophages induced by the clearance of apoptotic cells is called efferocytosis-induced macrophage proliferation (EIMP). Unlike macrophage proliferation caused by inflammation, it does not require IL-4 nor does it require LPS, IFN-γ, or colony-stimulating factor 1 (CSF1), and thus, it promotes the specific expansion of macrophages and forms an efferocytosis-tissue protection positive feedback loop (70).

#### 1.4.3 IRF5

**Figure 2:**
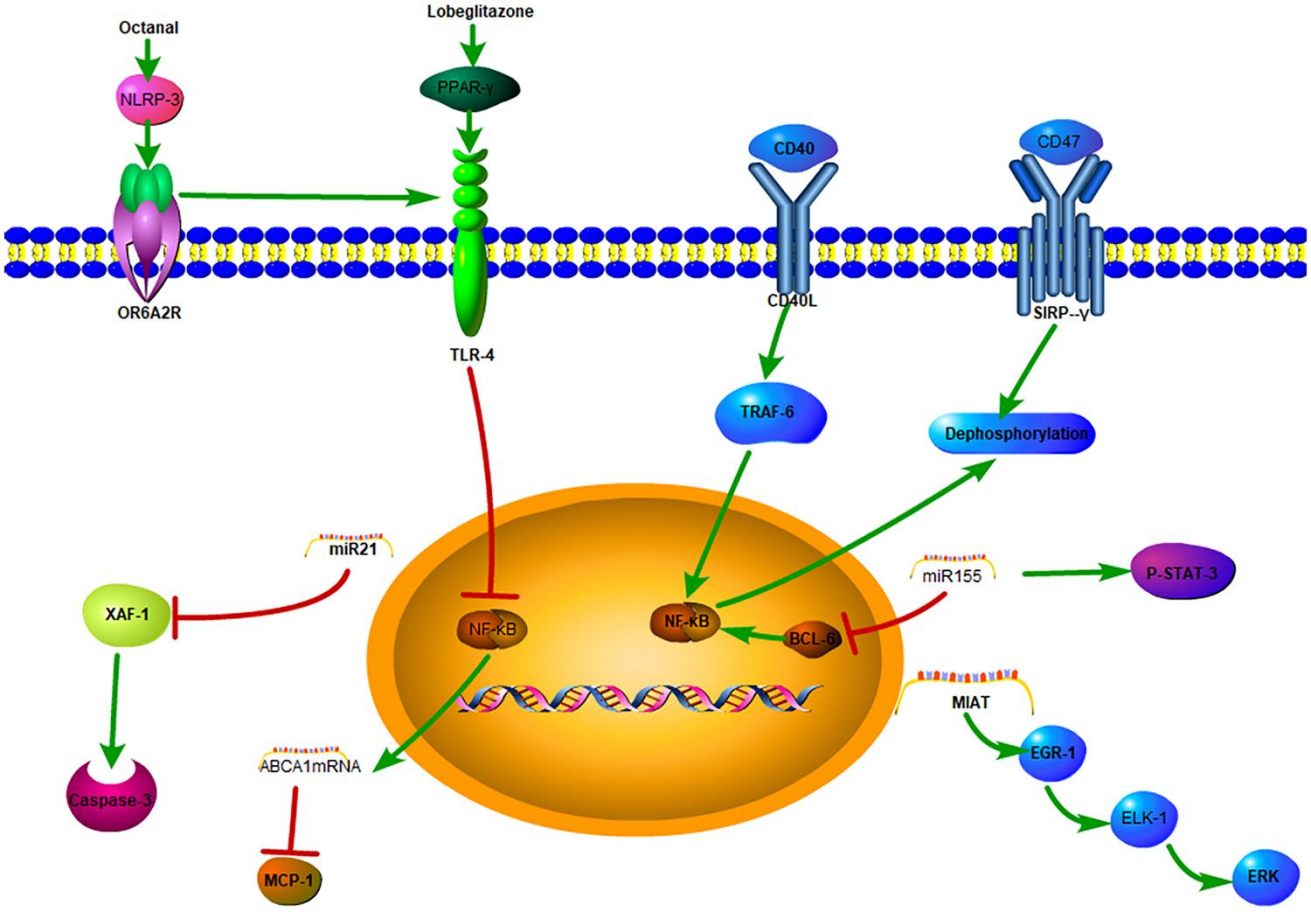
The mechanisms of macrophages in atherosclerosis.

Macrophages are core participants in CVDs, exerting both pro-atherogenic and anti-atherogenic effects (71). Previous studies have found that interferon regulatory factor 5 (IRF5) plays important roles in modulating pro-inflammatory macrophages in mice (72, 73). Edsfeldt et al. used human carotid endarterectomy and mouse carotid plaque-induced rupture models to show that IRF5 promotes macrophage activation and inhibits efferocytosis in plaques by producing chemokines CCL2 and CCL4, as well as high expression of integrin CD11c. This is followed by activation of inflammatory pathways, and then the resultant atheroma plaque has a larger necrotic core, which increases plaque vulnerability (71). Accordingly, restoring the transcriptional state of macrophages is an attractive strategy for the treatment of CVDs. Macrophages play key role in the pathogenesis of atherosclerosis, which functioning as instigators of inflammation and modulators of plaque stability (Figure 2). Developing novel therapeutic strategies targeting macrophages in atherosclerosis performs powerful potential for clinical therapy.

### 1.5 New advances in macrophage-targeted therapy

#### 1.5.1 Inhibition of inflammatory cytokines

Targeting *OR6A2* and other olfactory receptors (OLFRs) by inhibiting the NLRP3 inflammasome to reduce the production of pro-inflammatory cytokines may represent a new therapeutic strategy for preventing and reversing atherosclerosis (36). A novel near-infrared–emitting PPARγ activator developed as the therapeutic nano-targeted drug MMR-Lobe-Cy7 (mannose-polyethylene glycol-ethylene glycol chitosan-deoxycholic acid-cyano 7-rosiglitazone) has been shown to specifically target macrophages in the atherosclerotic plaque area, reducing the inflammatory response, increasing collagen content, and transforming an inflammatory plaque into a stable phenotype, and thus, offers a promising therapeutic method for controlling high-risk plaques in the acute phase (43).

#### 1.5.2 Immunomodulatory therapy

Immunomodulatory therapy shows great potential in the treatment of atherosclerosis. A macrophage-specific nanotherapy based on single-walled carbon nanotubes carrying chemical inhibitors of the anti-phagocytic cell CD47-signal regulatory protein α (SIRPα) signaling axis was shown to specifically regulate macrophages in atherosclerotic plaques, to reduce the inflammatory response, and to promote the clearance of apoptotic cells, making it a promising therapeutic strategy (40). In addition, a small molecular inhibitor of TRAF6 known as TRAF-STOP blocks the binding of CD40 and TRAF6, and packaging of TRAF-STOP in recombinant HDL nanoparticles was shown to be beneficial to the absorption of macrophages, to reduce the infiltration of macrophages, and to delay the process of atherosclerosis (46).

#### 1.5.3 RNA expression regulation

The polarization of macrophages and their participation in the inflammatory response are regulated by miRNAs (55), and this regulation in turn affects the process of atherosclerosis by regulating inflammatory protein expression, cholesterol transport, apoptosis, and clearance. For example, with the regulation of miR21 expression, the mismatch between apoptosis and clearance related to molecular clock expression in lesion macrophages is precisely regulated, thereby reducing the vulnerability of plaques in the morning (52). Studies have shown that CLA in polarized macrophages inhibits miR155 expression by upregulating the STAT-3/IL-10 signaling pathway, and this is expected to reverse atherosclerosis (56). Targeting MIAT also may become a new molecular therapeutic strategy to limit the progression of vascular inflammation and atherosclerosis under specific conditions (64). In addition, regulation of the NFATc3/miR-204 axis offers a promising therapeutic direction for the treatment of atherosclerotic diseases.

In summary, macrophages play an important role in the occurrence, progression, and regression of atherosclerosis. An in-depth understanding of their roles and comprehensive identification of the source, polarization, intercellular communication, and molecular regulation mechanisms of macrophages in plaques will allow a more complete understanding of why and how atherosclerosis occurs, guiding the design of new therapeutic directions for the treatment of CVDs and specifically revealing new targets for the prevention and treatment of CVDs to achieve clinical precise targeted therapy. Accordingly, research related to interventions targeting macrophage-related inflammation, efferocytosis, and lipid metabolism regulation is increasing. Future research is warranted to investigate the clinical application of macrophage-targeted therapies and their ability to improve CVD treatment and improve the prognosis of patients.
